# Sugar signals from oral glucose transporters elicit cephalic-phase insulin release in mice

**DOI:** 10.1186/s12576-023-00875-3

**Published:** 2023-07-31

**Authors:** Mitsuhito Takamori, Yoshihiro Mitoh, Kengo Horie, Masahiko Egusa, Takuya Miyawaki, Ryusuke Yoshida

**Affiliations:** 1 Department of Oral Physiology, Graduate School of Graduate School of Medicine, Dentistry and Pharmaceutical Sciences, Okayama University, Okayama, 700-8525 Japan; 2 Department of Dental Anesthesiology and Special Care Dentistry, Graduate School of Medicine, Dentistry and Pharmaceutical Sciences, Okayama University, Okayama, Japan; 3 Faculty of Medicine, Dentistry and Pharmaceutical Sciences, Okayama University, 2-5-1, Shikata-Cho, Kita-Ku, Okayama, 700-8525 Japan; 4 Advanced Research Center for Oral and Craniofacial Sciences, Okayama University Dental School, Okayama, Japan; 5 The Center for Special Needs Dentistry, Okayama University Hospital, Okayama, Japan

**Keywords:** Cephalic-phase insulin response, Glucose transporters, Glucose, Sweet taste receptor, Food intake

## Abstract

Cephalic-phase insulin release (CPIR) occurs before blood glucose increases after a meal. Although glucose is the most plausible cue to induce CPIR, peripheral sensory systems involved are not fully elucidated. We therefore examined roles of sweet sensing by a T1R3-dependent taste receptor and sugar sensing by oral glucose transporters in the oropharyngeal region in inducing CPIR. Spontaneous oral ingestion of glucose significantly increased plasma insulin 5 min later in wild-type (C57BL/6) and T1R3-knockout mice, but intragastric infusion did not. Oral treatment of glucose transporter inhibitors phlorizin and phloretin significantly reduced CPIR after spontaneous oral ingestion. In addition, a rapid increase in plasma insulin was significantly smaller in WT mice with spontaneous oral ingestion of nonmetabolizable glucose analog than in WT mice with spontaneous oral ingestion of glucose. Taken together, the T1R3-dependent receptor is not required for CPIR, but oral glucose transporters greatly contribute to induction of CPIR by sugars.

## Introduction

Cephalic-phase responses are physiological responses to prepare for optimal digestion, absorption, and metabolism of nutrients. They are induced by sensory signals from the head area, including oral and pharynx regions, at the beginning of and during the initiation of food intake [18]. These responses include increases in saliva secretion, gastric-acid secretion, stomach motility, thermogenesis, and hormonal secretions [5, 19, 25]. Cephalic-phase insulin release (CPIR) is one such cephalic-phase response. In rodents and humans, food stimulation to the oropharynx region induces insulin release from the pancreas before blood glucose increases, which helps suppress postprandial increases in blood glucose [6, 12, 17, 21, 23]. For induction of CPIR, sensory input from the oropharynx region is required, and the most plausible signal is oral glucose, because oral sugar ingestion induces CPIR [12]. However, peripheral sensory systems required for induction of CPIR are not fully elucidated.

In the oral cavity, the T1R2 + T1R3 heterodimer functions as a sweet taste receptor detects not only sugars but also for various artificial sweeteners, such as saccharin and sucralose, and even some proteins, such as brazzein and monellin [22, 26]. Therefore, CPIR could be induced by artificial sweeteners if sensory signals depend on T1R2 + T1R3 to induce CPIR. However, a previous study demonstrated that glucose and glucose-containing sugars, but not artificial sweeteners, elicited CPIR in mice [11]. In addition, T1R3-knockout (KO) mice showed CPIR when they orally ingested sugar solutions [10]. These previous findings suggest that the T1R2 + T1R3 sweet receptor is not required for induction of CPIR.

For sugar detection in the oral cavity, glucose transporters may also play an important role. T1R3-KO mice showed severely diminished gustatory nerve responses to artificial sweeteners, but responses to sugars, especially glucose, remained [7]. Thus, T1R3-independent receptor systems for oral detection of sugars exist in mice. As other candidates for sugar sensors, glucose transporters (GLUTs) and sodium-glucose transporters (SGLTs) were expressed in gustatory tissues of rodents [28, 31]. Indeed, a recent study investigating gustatory nerve responses and uptake of a fluorescent glucose analog in taste cells demonstrated that SGLT1 and other GLUTs contribute to sugar sensing in mouse taste cells [30]. In addition, SGLT1-dependent sugar sensing was reported to be enhanced by adrenomedullin [14]. Glucose is metabolized in the cell to produce adenosine triphosphate (ATP) as a source for cellular energy. In the case of islet β-cells, ATP produced by metabolizing glucose inhibits ATP-sensitive potassium channels (K_ATP_ channels), leading to depolarization and secretion of insulin [2, 3, 20]. Taste cells also express K_ATP_ channels [31, 32], so these cells could be depolarized by uptake of glucose via GLUTs and/or SGLTs. Signals from these cells might contribute to eliciting CPIR since these signals are specific to existence of glucose in the oral cavity.

In this study, we investigated sensory mechanisms involved in sugar induced CPIR. We focused on the T1R3-dependent taste signals and glucose-transporter-dependent taste signals by using transgenic mice, pharmacological blockers, and nonmetabolizable glucose analog.

## Methods

### Ethics approval

All animal experiments were performed in accordance with the National Institutes of Health *Guide for the Care and Use of Laboratory Animals* and approved by the Committee for Laboratory Animal Care and Use and the local ethics committee at Okayama University, Japan.

### Animals

These experiments used male C57BL/6J wild-type (WT) mice (purchased from CLEA Japan, Tokyo, Japan) and mice lacking the *Tas1r3* gene but expressing green fluorescent protein (GFP) in cells that usually express T1R3 (T1R3-GFP-KO mice), generated by crossing T1R3-KO mice [7] and T1R3-GFP mice [8], both strains were originally generated at Mount Sinai Medical School from the C57BL/6J strain and is maintained in this background. In total, we used 52 WT mice and 15 T1R3-GFP-KO mice. WT mice were divided into six groups; oral ingestion of glucose solution (OG group, n = 12), intragastric infusion of glucose solution (IG group, n = 13), oral ingestion of glucose solution + oral treatment of glucose transporter inhibitors (OG + GTI group, n = 11), oral ingestion of glucose solution + oral treatment of saline (OG–GTI group, n = 6), *oral ingestion o*f methyl α-d-glucopyranoside (MDG) + intragastric infusion of glucose solution (OMDG + IG group, n = 5), and oral ingestion of glucose solution + intragastric infusion of glucose solution (OG + IG group, n = 5). T1R3-GFP-KO mice were divided into 2 groups: the OG group (n = 6) and the IG group (n = 9). All mice were maintained in a 12/12-h light/dark cycle and fed standard rodent chow (MF, Oriental Yeast Co., Tokyo, Japan). In experiments, all mice received 2 mg/g body weight of glucose. Animals were 8–20 weeks of age, weighing 21–25 g.

### Training

All mice were trained to drink solution (water or 2 M glucose) from a licking spout connected to a lick meter (Yutaka Electronics Co., Gifu, Japan). On day 1 of training, each animal was water deprived for 23 h and then placed in the test cage and given free access to deionized water during the 1-h session. Days 2–5 were training sessions: animals were trained to drink deionized water from a licking spout after 12 h of water deprivation. During training sessions, the number of licks and amount of water licked were recorded to calculate amount of water per lick for each mouse. Day 6 was a resting session without any treatment.

### Oral ingestion of glucose solution

In the OG group, at day 7 glucose solution was administered by spontaneous ingestion of glucose solution from a water spout. Before testing, mice were deprived water and food for 12 h and then allowed to lick 2 M glucose solution from a lick spout in test cage. Total amount of ingested glucose was adjusted to 2 mg/g body weight by using water per lick for each mouse to control the number of licks allowed for 2 M glucose. The amount of water per lick ranged from 0.51 to 1.01 μl, so each mouse was allowed to lick 2 M glucose solution 120–220 times, depending on their individual water per lick and weight (2 M glucose solution contains 360 μg/μl glucose). Maximal lick rate is about 80 licks/10 s in all mice tested; thus, licking time for each mouse was within 30 s.

### Intragastric infusion of glucose solution

In the IG group, at day 7 glucose solution was administered directly into the stomach. Before testing, mice were deprived water and food for 12 h. Then, the mouse was secured at the scruff of its neck, and a curved feeding needle (Natsume Seisakusho, Tokyo, Japan) was gently inserted directly into its stomach, where 2 M glucose solution was injected at 2 mg/g body weight; the amount of infused 2 M glucose solution was thus 120–140 μl for each mouse.

### Oral ingestion of glucose solution + oral treatment of glucose transporter inhibitors (or saline)

In the OG + GTI group, at day 7 glucose administration was the same as for the OG group. Before testing, mice were deprived water and food for 12 h. Then, cotton gauze soaked with a mixture of the glucose transporter inhibitors phlorizin (1 mM; Sigma Aldrich, MA, USA) and phloretin (1 mM; Sigma Aldrich) was applied to the mouse tongue for 5 min with securing at the scruff of its neck. After treatment with these inhibitors, mice were allowed to lick 2 M glucose solution in a weight-specific manner using the same method as for the OG group (2 mg/g body weight, 120–220 licks). For control group (OG–GTI group), saline was used instead of a mixture of the glucose transporter. Other procedures were same as OG + GTI group.

### Oral ingestion of MDG (or glucose) + intragastric infusion of glucose solution

In the OMDG + IG group, glucose solution was administered at day 7 directly into the mouse’s stomach after it had licked 0.5 M MDG solution. In the OG + IG group, glucose solution was administered at day 7 directly into the mouse’s stomach after it had licked 0.5 M glucose solution. Before testing, mice were deprived water and food for 12 h. Then mice were allowed to lick 0.5 M MDG solution (OMDG + IG group) or 0.5 M glucose solution (OG + IG group) from a lick spout in a test cage, with the number of licks adjusted the same as for the OG group (120–220 licks). Immediately after mice had licked 0.5 M MDG solution or 0.5 M glucose solution, 2 M glucose solution was administered using the same method as for the IG group (total 2 mg glucose/g body weight).

### Blood glucose and plasma insulin measurements

At the beginning of each trial, mice were weighed and a baseline blood sample (0 min) was obtained from the tail vein. Then blood samples were collected from the tail vein 5, 10, 30, 60, and 90 min after the first lick of glucose solution (OG, OG + GTI, and OG–GTI groups) or intragastric infusion of glucose solution (IG, OMDG + IG, OG + IG groups). For blood glucose measurement, a single drop of tail blood at each time point was used to measure plasma glucose by a hand-held glucometer (Glutest Mint II, Sanwa Kagaku Kenkyusho, Aichi, Japan). For plasma insulin measurement, about 30 μl blood was collected at each time point in an EDTA-coated capillary tube (Paul Marienfeld, Baden-Württemberg, Germany). Collected samples were immediately centrifuged at 4700*g* for 3 min. Then plasma was obtained and was stored at − 80 °C until analysis. The stored samples were analyzed by Mouse/Rat Insulin ELISA Kit (Morinaga, Kanagawa, Japan) following the manufacturer’s instructions. Some ELISA data were excluded from analysis because of errors in calibration curve or in measurements.

### Statistical analysis

Differences among genotypes or groups and time were statistically analyzed by repeated two-way ANOVA. Differences in plasma glucose and insulin levels at each time point were statistically analyzed by Student's t-test with Bonferroni correction. Increases in plasma insulin level at 5 min were statistically analyzed by paired t-test by comparing plasma insulin levels at 0 min and 5 min. Differences in changes in insulin at 5 min were statistically analyzed by Student's t-test.

All statistical analyses were performed using Jamovi software (ver. 2.3.21, https://www.jamovi.org/). P-values < 0.05 were considered significant.

## Results

### Contribution of T1R3 to cephalic-phase insulin release

**Fig. 1:**
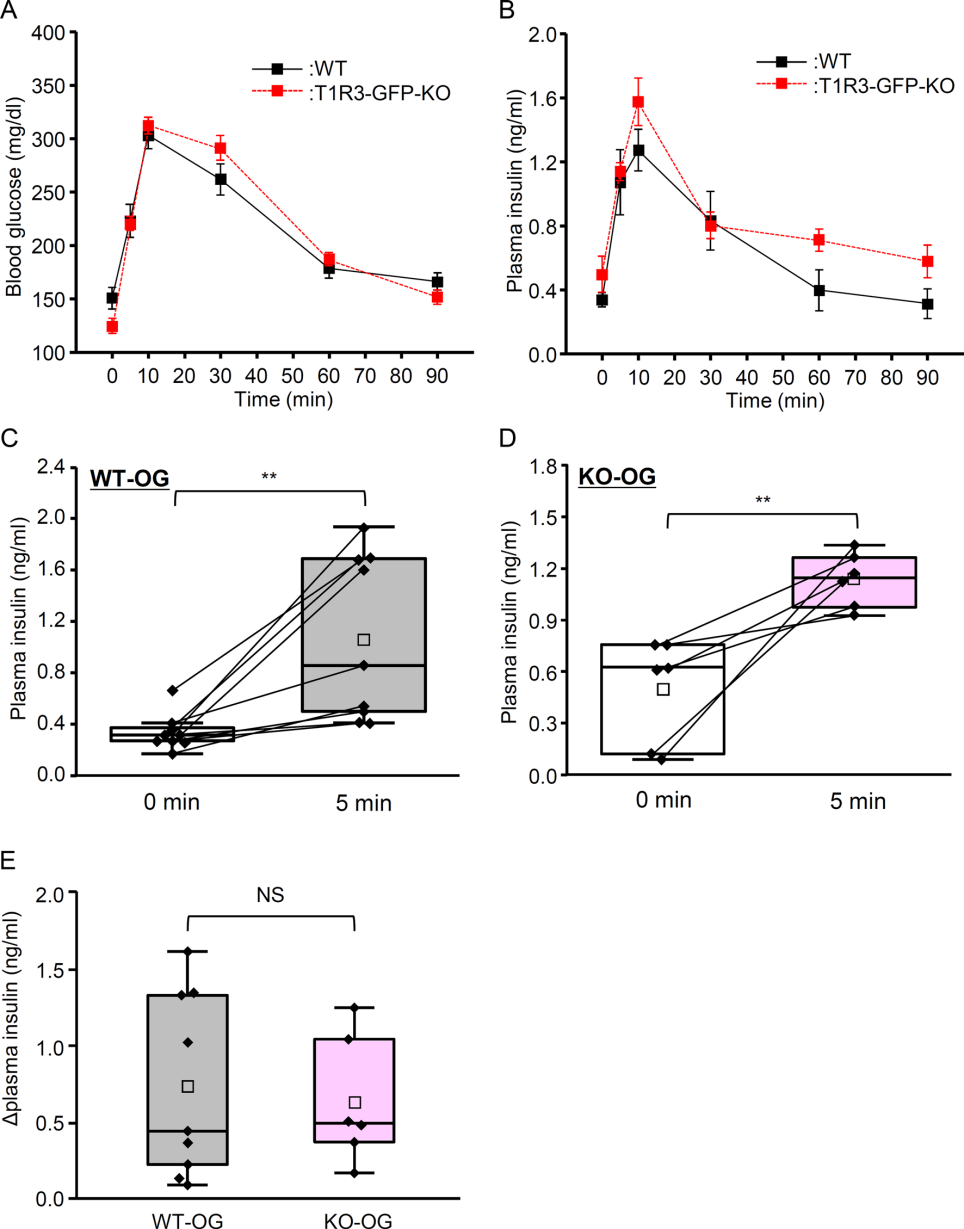
Blood glucose and plasma insulin levels after oral ingestion of glucose in wild-type and T1R3-GFP-knockout mice. **A** Blood glucose levels before and 5, 10, 30, 60, and 90 min after oral ingestion of glucose (2 mg/g body weight) in wild-type (WT) mice (n = 12) and T1R3-GFP-knockout (KO) mice (n = 6). **B** Plasma insulin levels before and 5, 10, 30, 60, and 90 min after oral ingestion of glucose (2 mg/g body weight) in WT mice (n = 9) and T1R3-GFP-KO mice (n = 6). Data are presented as the mean ± standard error. **C**, **D** Plasma insulin levels at 0 and 5 min after oral glucose (OG) ingestion (2 mg/g body weight) in WT mice (**C**) and T1R3-GFP-KO mice (**D**). **E** Changes in plasma insulin levels 5 min after glucose ingestion for WT and T1R3-KO mice. In these box plots, the box indicates the 25th and 75th percentiles; line across the box, the median; square, the mean; and whiskers, maximum and minimum values. Each point indicates individual data. **P < 0.01, paired t-test. *NS* not significantly different, Student’s t-test

We first tested whether WT and T1R3-GFP-KO mice had different temporal dynamics of plasma insulin and blood glucose after consuming glucose solution by oral ingestion (OG group) or intragastric infusion (IG group). Our previous report demonstrated that T1R3-GFP-KO mice have diminished oral sensitivity to sugars compared to WT mice in short-term lick tests with a sweet-bitter mixture paradigm [29]. In the OG group, both WT and T1R3-GFP-KO mice showed a rapid increase in blood glucose levels, which reached maximum at 10 min (Fig. 1A). Plasma insulin levels reached maximum at 10 min in WT mice and T1R3-GFP-KO mice (Fig. 1B). Comparison between WT and T1R3-GFP-KO mice showed no significant main effect of genotype on blood glucose level (Fig. 1A, Table 1; P = 0.995, repeated two-way ANOVA) and plasma insulin level (Fig. 1B, Table 1; P = 0.295, repeated two-way ANOVA). As an indicator of the occurrence of CPIR, we used increase in plasma insulin level at 5 min after glucose intake/administration [10, 11]. In the OG group, for both WT and T1R3-GFP-KO mice plasma insulin level at 5 min was significantly greater than at baseline (0 min, Fig. 1C, D; P < 0.01, paired t-test). Changes in plasma insulin level 5 min after ingestion of glucose were not significantly different between WT and T1R3-GFP-KO mice (Fig. 1E; P > 0.1, Student’s t-test).

**Table 1:** Summary of ANOVA results

	Measurement	Variation	F value	df	P value
B6, OG vs IG	Plasma insulin	Time	20.55	5	< 0.001***
		Treatment	0.023	1	0.883
		Interaction	2.78	5	0.024*
	Blood glucose	Time	50.92	5	< 0.001***
		Treatment	0.117	1	0.736
		Interaction	9.74	5	< 0.001***
T1R3-GFP-KO, OG vs IG	Plasma insulin	Time	24.11	5	< 0.001***
		Treatment	0.383	1	0.550
		Interaction	3.21	5	0.014*
	Blood glucose	Time	54.64	5	< 0.001***
		Treatment	1.66	1	0.219
		Interaction	1.73	5	0.140
OG, B6 vs T1R3-GFP-KO	Plasma insulin	Time	36.81	5	< 0.001***
		Species	1.19	1	0.295
		Interaction	1.11	5	0.364
	Blood glucose	Time	103.36	5	< 0.001***
		Species	0.00	1	0.995
		Interaction	2.19	5	0.063
IG, B6 vs T1R3-KO	Plasma insulin	Time	6.65	5	< 0.001***
		Species	0.325	1	0.578
		Interaction	1.14	5	0.346
	Blood glucose	Time	40.5	5	< 0.001***
		Species	1.9	1	0.184
		Interaction	1.56	5	0.179
B6, OG–GTI vs OG + GTI	Plasma insulin	Time	7.441	5	< 0.001***
		Treatment	0.657	1	0.433
		Interaction	0.723	5	0.609
	Blood glucose	Time	76.2	5	< 0.001***
		Treatment	6.23	1	0.025
		Interaction	10.6	5	< 0.001***
B6, OG + IG vs OMDG + IG	Plasma insulin	Time	11.655	5	< 0.001***
		Treatment	5.05	1	0.055
		Interaction	0.200	5	0.961
	Blood glucose	Time	57.36	5	< 0.001***
		Treatment	0.896	1	0.733
		Interaction	1.50	5	0.213

**Fig. 2:**
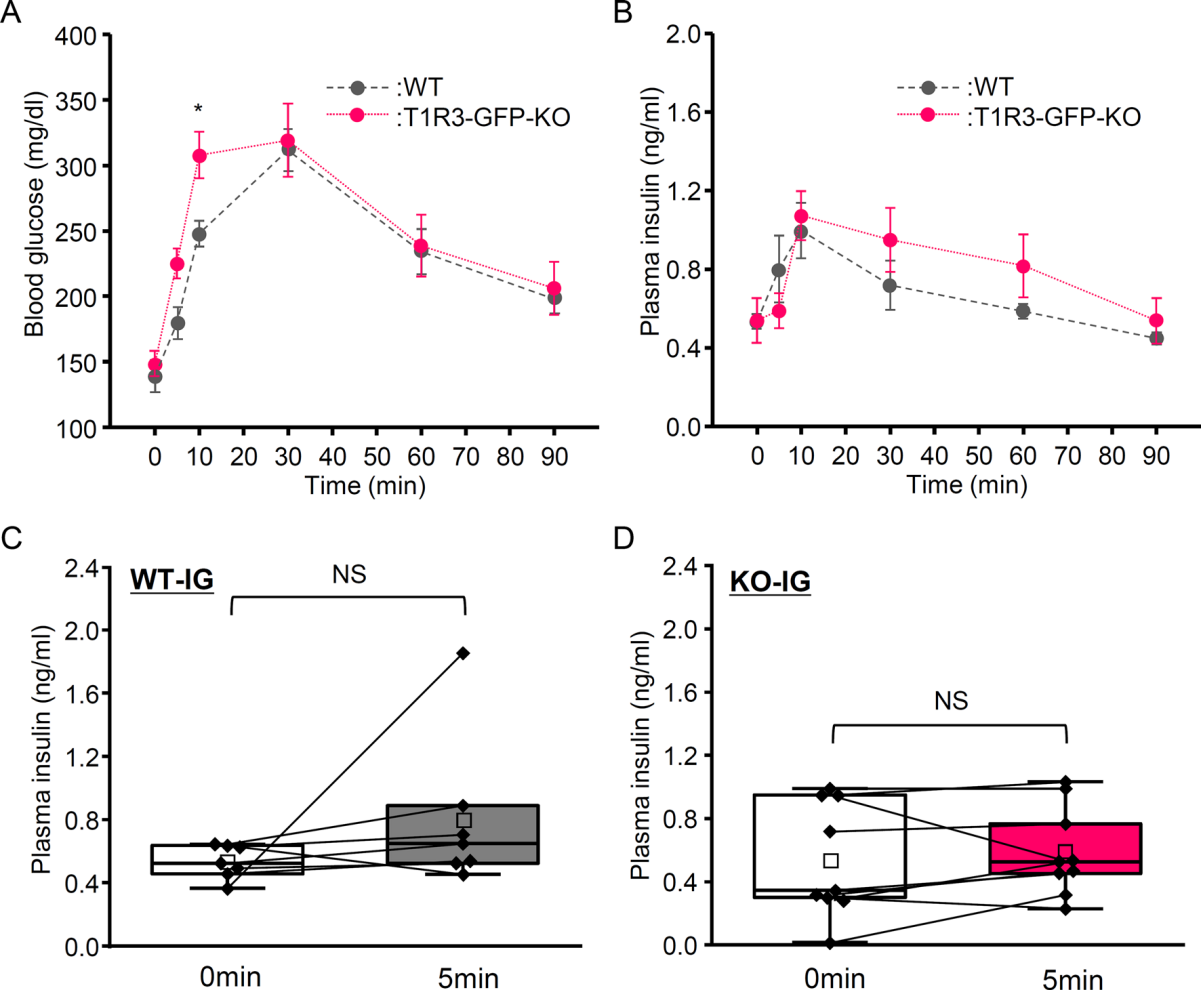
Blood glucose and plasma insulin levels after intragastric infusion of glucose in wild-type and T1R3-GFP-knockout mice. **A** Blood glucose levels before and 5, 10, 30, 60, and 90 min after intragastric infusion of glucose (2 mg/g body weight) in wild-type (WT) mice (n = 13) and T1R3-GFP-knockout (KO) mice (n = 9). **B** Plasma insulin levels before and 5, 10, 30, 60, and 90 min after intragastric infusion of glucose (2 mg/g body weight) in WT mice (n = 7) and T1R3-GFP-KO mice (n = 9). *P < 0.05, Student’s t-test with Bonferroni correction (WT vs. T1R3-GFP-KO). Data are presented as the mean ± standard error. **C**, **D** Plasma insulin levels at 0 and 5 min after intragastric infusion (IG) of glucose (2 mg/g body weight) in WT mice (**C**) and T1R3-GFP-KO mice (**D**). Box plots are as in Fig. 1E. Each point indicates individual data. *NS* no significant difference, paired t-test

On the other hand, the IG group showed more prolonged increase in blood glucose level in both WT and T1R3-GFP-KO mice (Fig. 2A). Blood glucose levels reached maximum at 30 min and plasma insulin level reached maximum at 10 min in for both WT and T1R3-GFP-KO mice (Fig. 2A, B). Comparison between WT and T1R3-GFP-KO mice showed no significant main effect of genotype in either blood glucose level (Fig. 2A, Table 1; P = 0.184, repeated two-way ANOVA) or plasma insulin level (Fig. 2B, Table 1; P = 0.578, repeated two-way ANOVA). Blood glucose level of T1R3-GFP-KO mice was significantly greater than that of WT mice at 10 min (P < 0.05, Student’s t-test with Bonferroni correction). In both WT and T1R3-GFP-KO mice of the IG group, plasma insulin level was not significantly different between 0 and 5 min (Fig. 2C, D; P > 0.1; paired t-test). These results suggest that CPIR was induced by oral glucose ingestion but not by intragastric administration of glucose solution in both WT and T1R3-GFP-KO mice.

### Contribution of oral glucose transporters to cephalic-phase insulin release

**Fig. 3:**
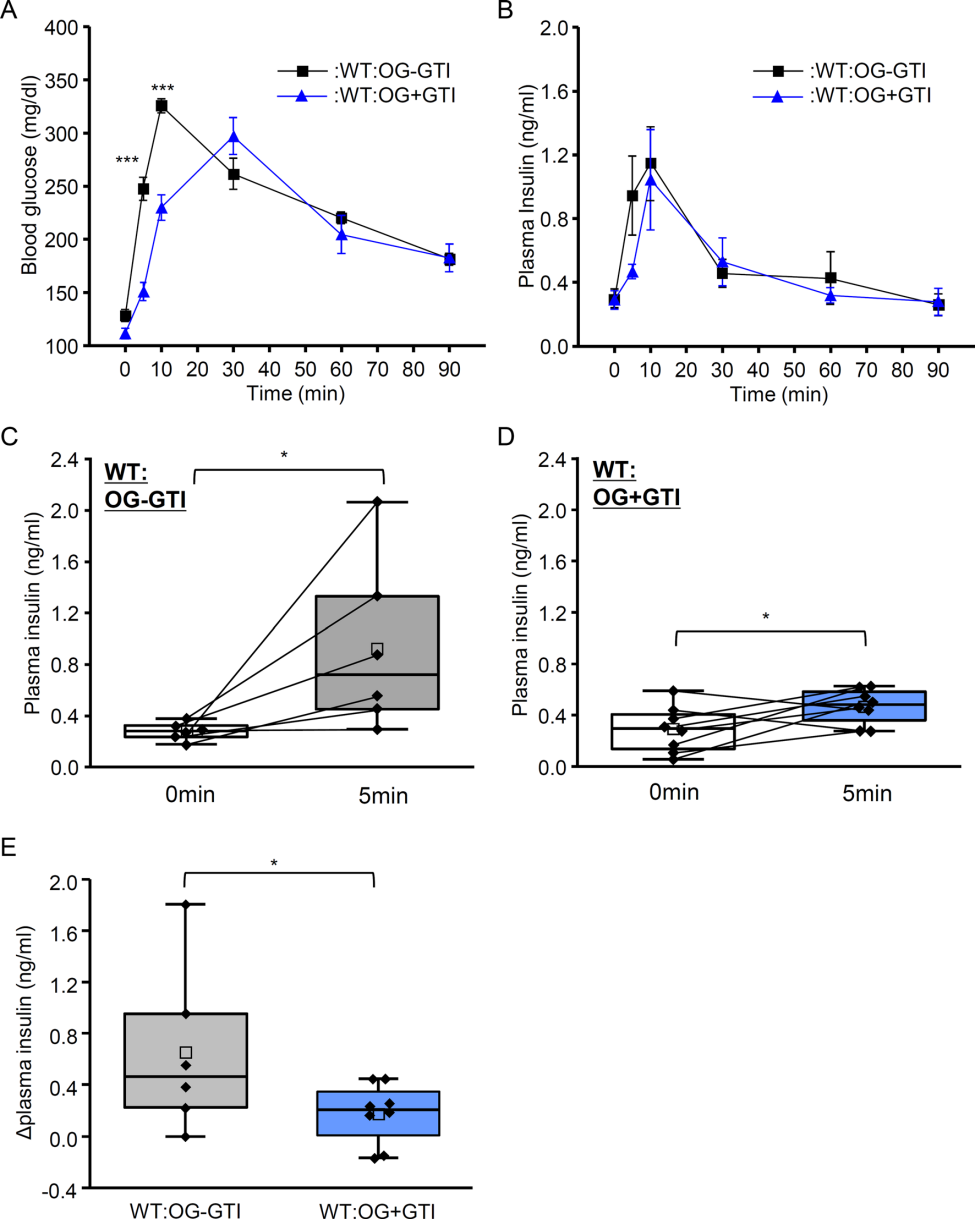
Blood glucose and plasma insulin levels after oral ingestion of glucose with glucose transporter oral treatment. **A** Blood glucose levels before and 5, 10, 30, 60, and 90 min after oral ingestion of glucose after oral treatment with saline (OG–GTI; n = 6) or 1 mM phlorizin plus 1 mM phloretin (OG + GTI; n = 11). **B.** Plasma insulin levels before and 5, 10, 30, 60, and 90 min after OG–GTI (n = 6) and OG + GTI (n = 8) protocols. All mice received 2 mg/g body weight of glucose. ***P < 0.05, Student's t-test with Bonferroni correction. Data are presented as the mean ± standard error. **C** Plasma insulin levels at 0 and 5 min after the OG–GTI protocol. *P < 0.05, paired t-test. **D** Plasma insulin levels at 0 and 5 min after the OG + GTI protocol. *P < 0.05, paired t-test. **E** Increases in plasma insulin levels at 5 min for the OG–GTI and OG + GTI groups. *P < 0.05, Student's t-test. Box plots are as in Fig. 1E. Each point indicates individual data

A rapid increase in plasma insulin level was observed in the OG group for T1R3-GFP-KO mice. Therefore, T1R3-dependent receptors may not be involved in induction of CPIR. Previous studies demonstrated that glucose transporters were expressed in taste bud cells [28, 31]. In addition, these glucose transporters may have important roles in detection of oral sugars [30]. Therefore, we sought to determine if glucose transporters in oral cavity could contribute to induction of CPIR. We used a mixture of phlorizin and phloretin to inhibit both SGLTs and GLUTs, respectively. These inhibitors were applied only to the oral cavity of WT mice before oral ingestion of glucose solution (OG + GTI group). As control, saline was used instead of treatment of these inhibitors (OG–GTI group). The time course of blood glucose changes in the OG + GTI group was similar to that in the IG group, and blood glucose level reached maximum at 30 min (Fig. 3A). Comparison of WT mice in OG–GTI and OG + GTI groups showed a significant main effect of group in blood glucose level (Fig. 3A, Table 1; P = 0.025, repeated two-way ANOVA), but no significant main effect of group in plasma insulin level (Fig. 3B, Table 1; P = 0.433, repeated two-way ANOVA). Blood glucose level of OG + GTI was significantly smaller than that of OG–GTI group at 5 and 10 min (P < 0.001, Student’s t-test with Bonferroni correction). Same as OG group, OG–GTI group showed significant difference of plasma insulin level between 0 and 5 min (Fig. 3C). In the OG + GTI group, plasma insulin level at 5 min was significantly different from that at 0 min (Fig. 3D; P < 0.05, paired t-test), but the increase was significantly smaller than that in the OG–GTI group (Fig. 3E; P < 0.05, Student's t-test). These results suggest that oral treatment of glucose transporter inhibitors inhibits the rapid increase in plasma insulin level after oral ingestion of glucose solution, yet a small increase in plasma insulin remains.

### MDG did not induce cephalic-phase insulin release

**Fig. 4:**
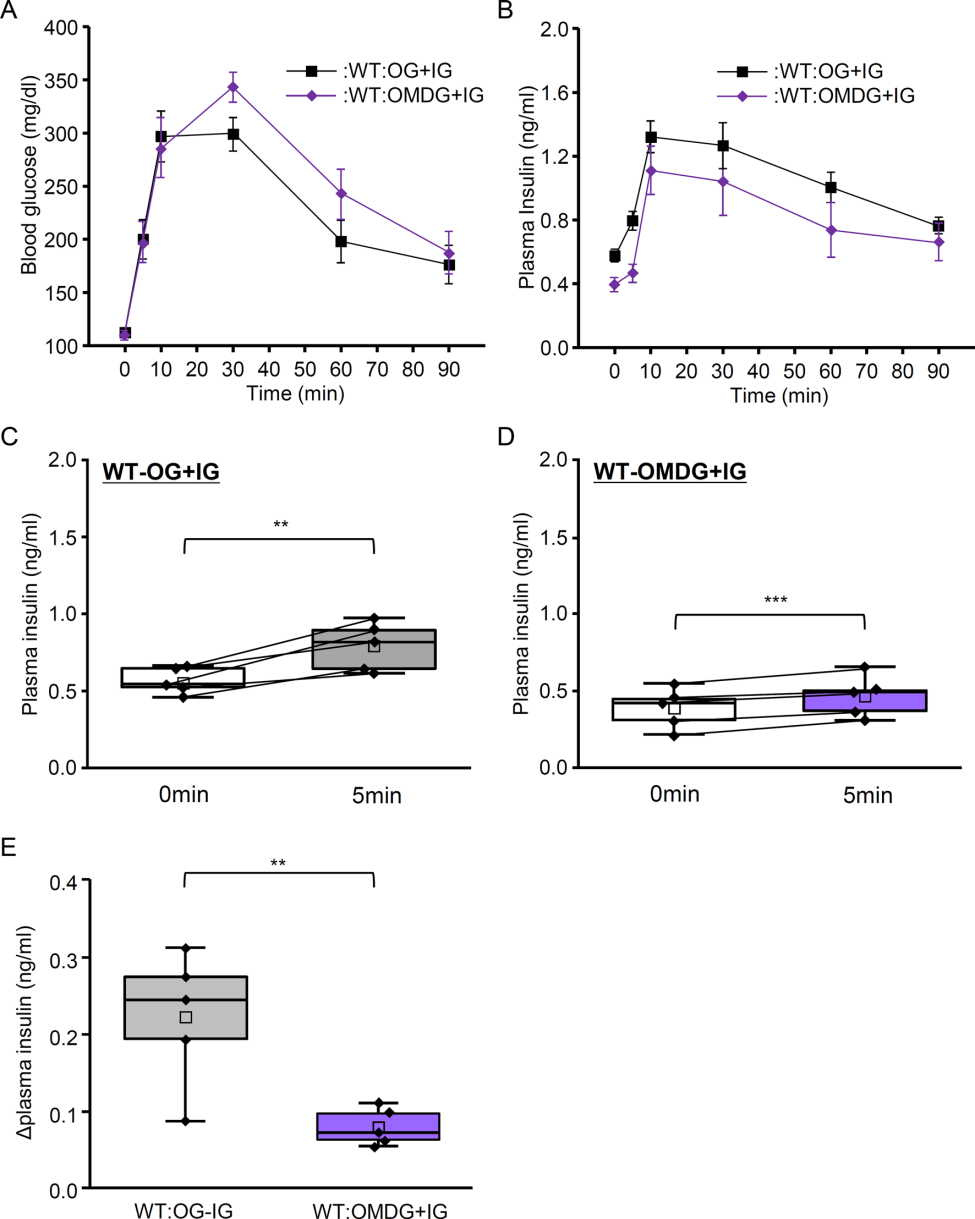
Blood glucose and plasma insulin levels after MDG ingestion. **A** Blood glucose levels before and 5, 10, 30, 60, and 90 min after oral ingestion of 0.5 M glucose solution with intragastric infusion of glucose (OG + IG; n = 5) and oral ingestion of 0.5 M MDG solution with intragastric infusion of glucose (OMDG + IG; n = 5). **B** Plasma insulin levels before and 5, 10, 30, 60 and 90 min after OG + IG (n = 5), and OMDG + IG (n = 5) protocols. All mice received 2 mg/g body weight of glucose. Data are presented as the mean ± standard error. **C** Plasma insulin levels at 0 and 5 min after OG + IG protocol (2 mg/g body weight). **P < 0.01, paired t-test. **D** Plasma insulin levels at 0 and 5 min after OMDG + IG protocol (2 mg/g body weight). ***P < 0.001, paired t-test. **E** Increase in insulin concentration at 5 min for OG + IG and OMDG + IG groups. **P < 0.01, Student’s t-test. Box plots are as in Fig. 1E. Each point indicates individual data

MDG is non-metabolizable glucose analog, which can pass glucose transporters. To test whether activation of glucose transporters on the tongue itself is able to induce CPIR in mice, we measured blood glucose level and plasma insulin level of WT mice after spontaneous drinking of MDG solution. To standardize glucose administration, mice were given intragastric infusion of glucose (2 mg/g body weight) immediately after drinking MDG solution (OMDG + IG group). As control, mice were given intragastric infusion of glucose (1.5 mg/g body weight) immediately after drinking 0.5 M glucose solution (0.5 mg glucose/g body weight, OG + IG group). OMDG + IG group showed maximum blood glucose level at 30 min (Fig. 4A). Comparison of WT mice between OG + IG and OMDG + IG groups showed no significant main effect of group in blood glucose level (Fig. 4A, Table 1; P = 0.0.213, repeated two-way ANOVA), and plasma insulin level reached maximum at 10 min in both groups (Fig. 4B); we found no significant main effect of group in plasma insulin level (Fig. 4B, Table 1; P = 0.055, two-way ANOVA). Same as OG group, OG + IG group showed significant difference of plasma insulin level between 0 and 5 min (Fig. 4C; P < 0.01, paired t-test). In the OMDG + IG group, plasma insulin level at 5 min was significantly different from that at 0 min (Fig. 4D; P < 0.001, paired t-test), but the increase was significantly smaller than that in the OG + IG group (Fig. 4E; P < 0.01, Student's t-test). These results suggest that MDG do not induce rapid increase in plasma insulin level like glucose.

## Discussion

Since the sweet taste receptor T1R2 + T1R3 plays a critical role in detecting sweet compounds, including sugars, artificial sweeteners, and some proteins, we first focused on the role of T1R3-dependent signals on induction of CPIR. Previous studies demonstrated that the early rise in plasma insulin reflected a cephalic-phase response [10, 11]. Therefore, we inferred that a significant increase in blood insulin level 5 min after glucose administration signaled CPIR. In both WT and T1R3-GFP-KO mice, those that consumed glucose solution orally (OG group), but not those that received glucose intragastrically (IG group), showed significantly increased plasma insulin 5 min after glucose administration (see Figs. 1, 2), with similar changes observed in WT and T1R3-GFP-KO mice (Fig. 1E). These results suggest that the T1R3-dependent signal does not contribute to CPIR in mice. Our results were consistent with a previous study demonstrating that a T1R2 + T1R3-independent taste transduction pathway is not required for sugar-induced CPIR [10]. In mice, oral stimulation with artificial sweeteners such as saccharin, sucralose, acesulfame K, and SC45647 did not elicit CPIR [11]. Artificial sweeteners mainly activate T1R2 + T1R3 taste receptors in the oral cavity. Thus, these data support the hypothesis that activation of T1R2 + T1R3 receptor may not contribute to induction of CPIR in mice. However, other studies demonstrated that saccharin stimulation could induce CPIR in rats [4, 13, 27]. In humans, previous studies demonstrated conflicting results: some showed that saccharin and other artificial sweeteners could elicit CPIR [9, 16], but some did not [1, 24]. Differences may be attributed to different experimental procedures and subjects, such as how insulin was measured, how sweeteners were used for stimulation, and type of subject (normal vs. pathological). At least in mice, CPIR induced by artificial sweeteners has not been reported, and our and other groups’ data indicate that a T1R3-dependent receptor is not involved in eliciting CPIR. Collectively, there may be some species differences in the mechanisms for eliciting CPIR. Future studies may be required to understand the mechanisms for induction of CPIR by artificial sweeteners.

For sugar detection, not only T1R3-dependent receptors but also glucose transporters play an important role. In T1R3-KO mice, gustatory nerve responses to sugars, especially glucose, remained [7]. Such residual responses to sugars in T1R3-KO mice may be derived from glucose transporters, because some taste bud cells express glucose transporters [31], and phlorizin significantly inhibits gustatory nerve responses to glucose [30]. We hypothesized that glucose transporters on the tongue may function as glucose sensors to induce CPIR. Our result showed that oral treatment of the glucose transporter inhibitors phlorizin plus phloretin significantly reduced increases in plasma insulin 5 min after oral ingestion of glucose solution (Fig. 3), suggesting that oral glucose transporters contribute to eliciting CPIR. However, activation of glucose transporters itself may not induce CPIR since spontaneous drinking of nonmetabolizable glucose analog MDG solution did not increase plasma insulin level 5 min after MDG ingestion compared to its control group (Fig. 4). Therefore, metabolization of glucose after entering taste cells would be required for making neural signals contributing to induction of CPIR. Taste cells are able to uptake oral glucose via glucose transporters [30] to produce ATP. This process would inhibit K_ATP_ channels expressed in taste cells [31, 32], leading to depolarization of taste cells. Thus, not only glucose transporters but also K_ATP_ channels on the tongue may have critical functions in sensing glucose in the oral cavity. A previous study demonstrated that mice lacking SUR1, a K_ATP_ channel component, did not show CPIR [11]. Because impairment of either glucose transporters or K_ATP_ channels diminished CPIR, neural signals derived from activation of the glucose transporter–K_ATP_ channel pathway in taste cells might be necessary to induce CPIR in mice. These neural signals may be different, at least in some ways, from those derived from activation of T1R3-dependent receptors. At the gustatory nerve fiber level, sweet fibers were classified into three groups: T1R-dependent type, Glc-type, and mixed-type fibers [30]. Among them, Glc-type fibers might convey signals for induction of CPIR but not contribute to perception of sweet taste of sugars. Projection targets in the central nervous system also may be different among these three types of sweet fibers, and this should be examined in future studies.

We observed that blood glucose level of T1R3-GFP-KO mice was significantly greater than that of WT mice at 10 min when glucose was administrated intragastrically (Fig. 2A). We did not find such difference when mice ingested glucose solution orally (Fig. 1A). Impaired glucose tolerance in T1R3-KO mice after intragastric infusion of glucose has been reported in previous study [10]. In addition, gustducin-KO mice showed higher plasma glucose concentrations after gavage- administration of glucose than WT mice [15]. Gustducin has a crucial role in intracellular signaling pathway after activation of T1R2/T1R3 receptor. Thus, lack of T1R3 signaling may lead to impaired glucose tolerance of mice if glucose is directly infused into gut. In case of oral intake of glucose solution, T1R3-KO mice did not show such impaired glucose tolerance in this (Fig. 1A) and previous study [10]. Therefore, CPIR induced by oral glucose signal could contribute to better glucose tolerance. Since T1R3-KO mice showed impaired glucose tolerance after gavage- administration of glucose, T1R3-dependent signals in the gastrointestinal tract may improve the glucose tolerance in mice. Further studies are required to test this possibility.

In this study, we demonstrated that oral consumption of MDG solution with intragastric administration of glucose solution (OMDG + IG group) induced a significant increase in plasma insulin level 5 min after glucose administration, although this increase was smaller than that in the OG + IG group (Fig. 4). In addition, the OG + GTI group also showed a significant increase in plasma insulin level 5 min after glucose administration (Fig. 3C). Because the IG group did not show such an increase in plasma insulin levels, these results suggest a possibility that some somatosensory signals from the oropharynx region take part in eliciting CPIR. However, omission of chemical signals derived from glucose transporters on the tongue greatly affected the increase in plasma insulin level 5 min after glucose administration (Figs. 3, 4). Therefore, somatosensory signals may have a supportive role for, or a synergistic effect on, induction of CPIR if they have some roles in CPIR. Somatosensory signals from the oropharynx region propagate via the trigeminal nerve, the glossopharyngeal nerve, and the vagus nerve. Taste signals propagate via the facial nerve (the chorda tympani nerve and the greater petrosal nerve), the glossopharyngeal nerve, and the vagus nerve. These signals may be integrated in some nuclei in the central nervous system, such as nucleus of the solitary tract and the dorsal motor nucleus of the vagus nerve. Signal integration of different modalities would enhance efferent signals to β-cells in islets to induce CPIR. This possibility should be investigated in future studies.

## Conclusions

We have demonstrated that chemical signals from oral glucose transporters but not T1R3-dependent sweet taste receptors contribute to sugar induced CPIR in mice. In addition, metabolization of glucose after entering taste cells via glucose transporters might be important for making neural signals to induce CPIR. Thus, we first demonstrated that glucose transporters on the taste cells may have different functions from T1R3-dependent sweet taste receptors. Although neural signals derived from oral glucose transporters shape CPIR, signal integration of different modalities might be important for induction of large CPIR. That is, multiple neural signals elicited by ingestion of foods may be required for induction of proper CPIR to control glucose metabolism after food intake.

## Data Availability

All data supporting the findings of this study are available from the corresponding author upon request.

## Abbreviations

CPIR: Cephalic-phase insulin release
GLUT: Glucose transporter
OG: Oral ingestion of glucose solution
IG: Intragastric infusion of glucose solution
OG + GTI: Oral ingestion of glucose solution plus oral treatment of glucose transporter inhibitors
OW + IG: Oral ingestion of water plus intragastric infusion of glucose solution
KO: Knockout
SGLT: Sodium glucose cotransporter
T1R2 + T1R3: Heterodimer of *Tas1r2* and *Tas1r3* gene products
WT: Wild-type
GFP: Green fluorescent protein
